# Supplementary figures and images for: Alternative poly-adenylation modulates α1-antitrypsin expression in chronic obstructive pulmonary disease
Source: PLoS Genet. 2021 Nov 16;17(11):e1009912. doi: 10.1371/journal.pgen.1009912 (PMC8631626; doi:10.1371/journal.pgen.1009912)

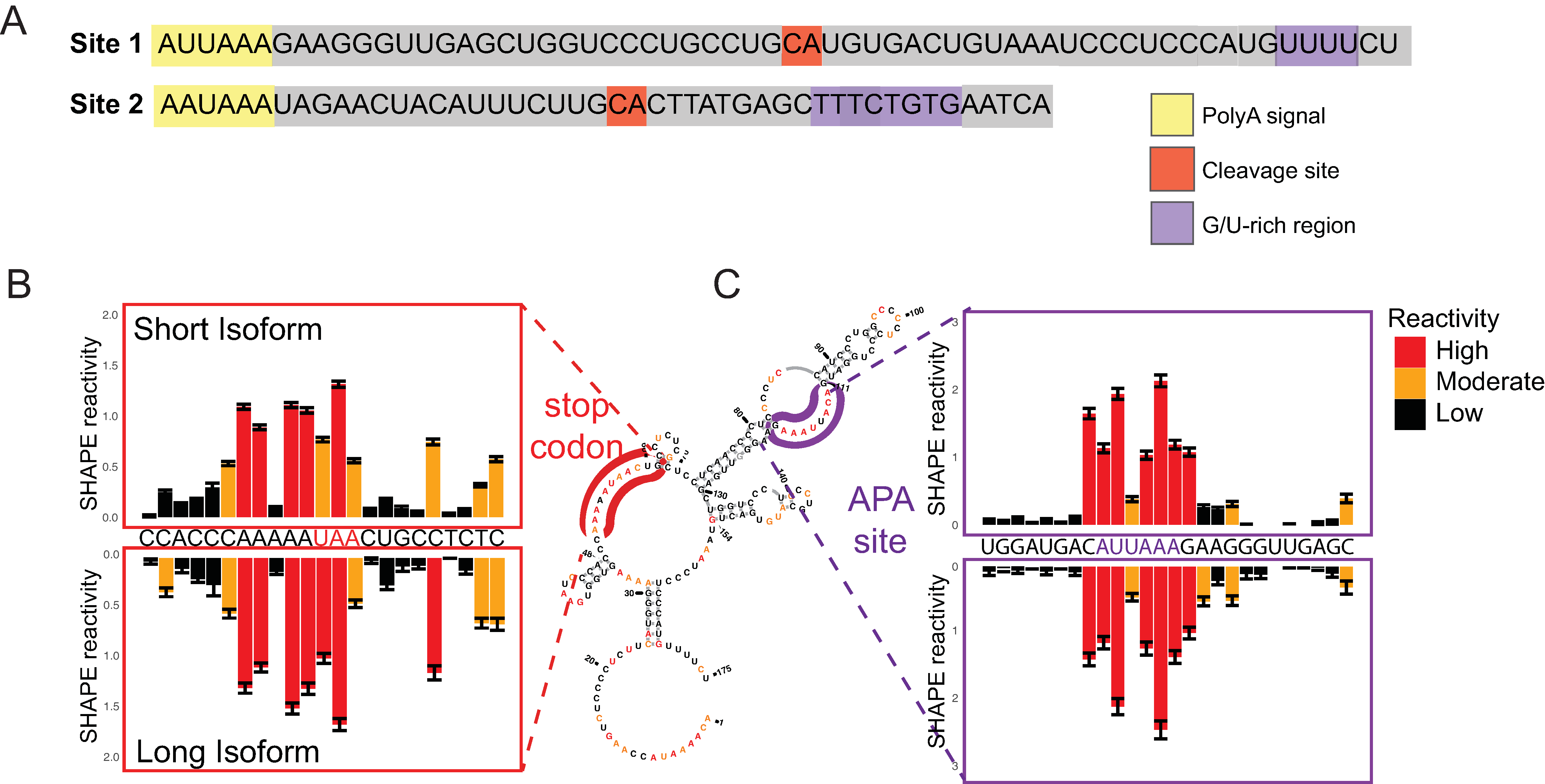

Supplement: S1 Fig — A) SERPINA1 mRNAS 3′ UTR sequence showing consensus polyA signal (yellow), cleavage site as determined by 3′ End sequencing (red) and putative G/U-rich region downstream of cleavage site. B) Structural analysis of proximal site in SERPINA1 mRNA 3′ UTR. We observe that both the stop codon (red) and APA site (purple) are highly accessible (i.e. have high SHAPE reactivity), allowing both these sites access to the Ribosome and cleavage machinery respectively. (TIF) [file pgen.1009912.s001.tif]

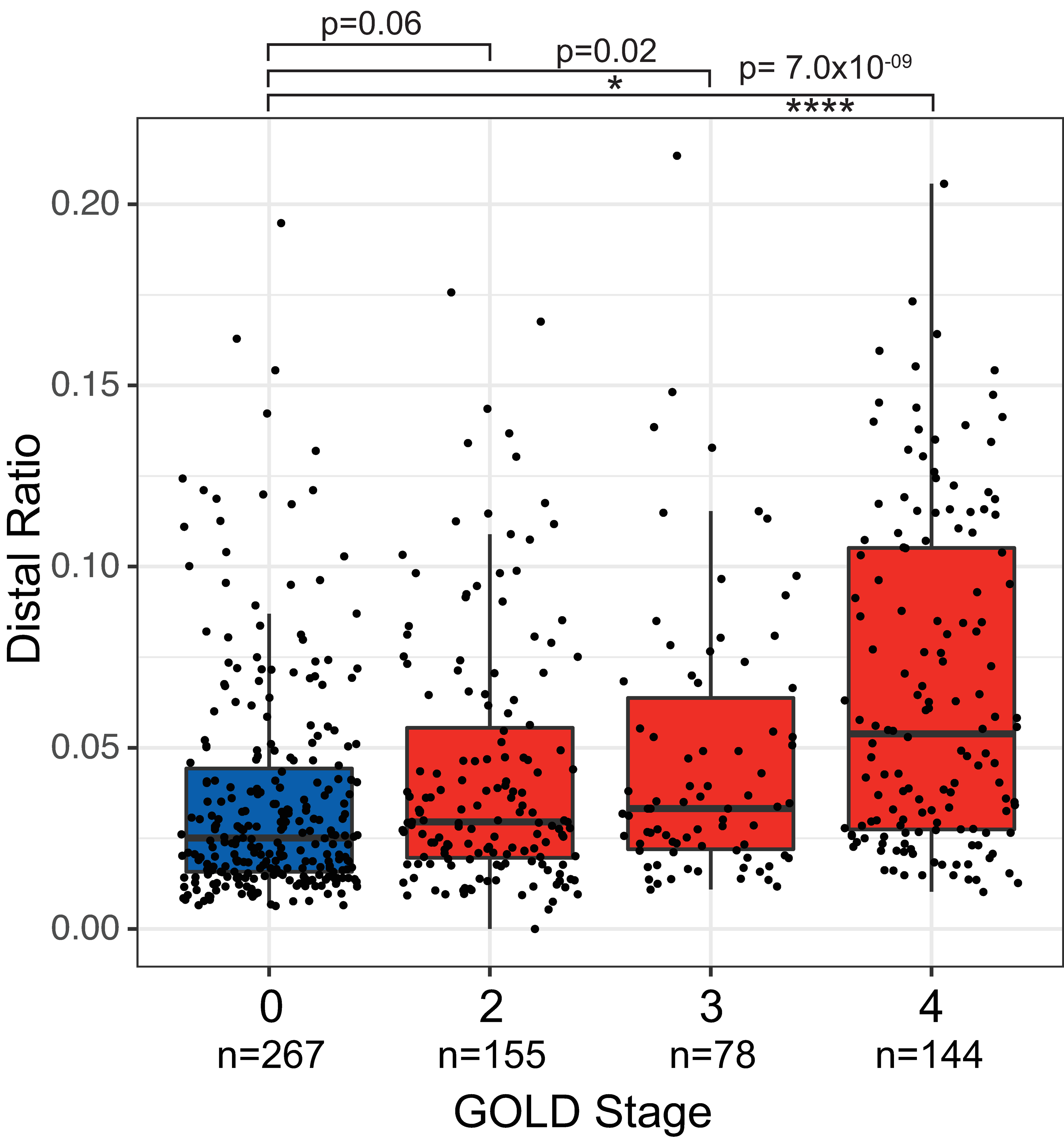

Supplement: S2 Fig — SERPINA1 3′ UTR distal ratio for disease severity as measured by GOLD stage rating of COPD (red) and non-COPD (blue) indicating an increase in distal ratio with increasing disease severity. (TIF) [file pgen.1009912.s002.tif]

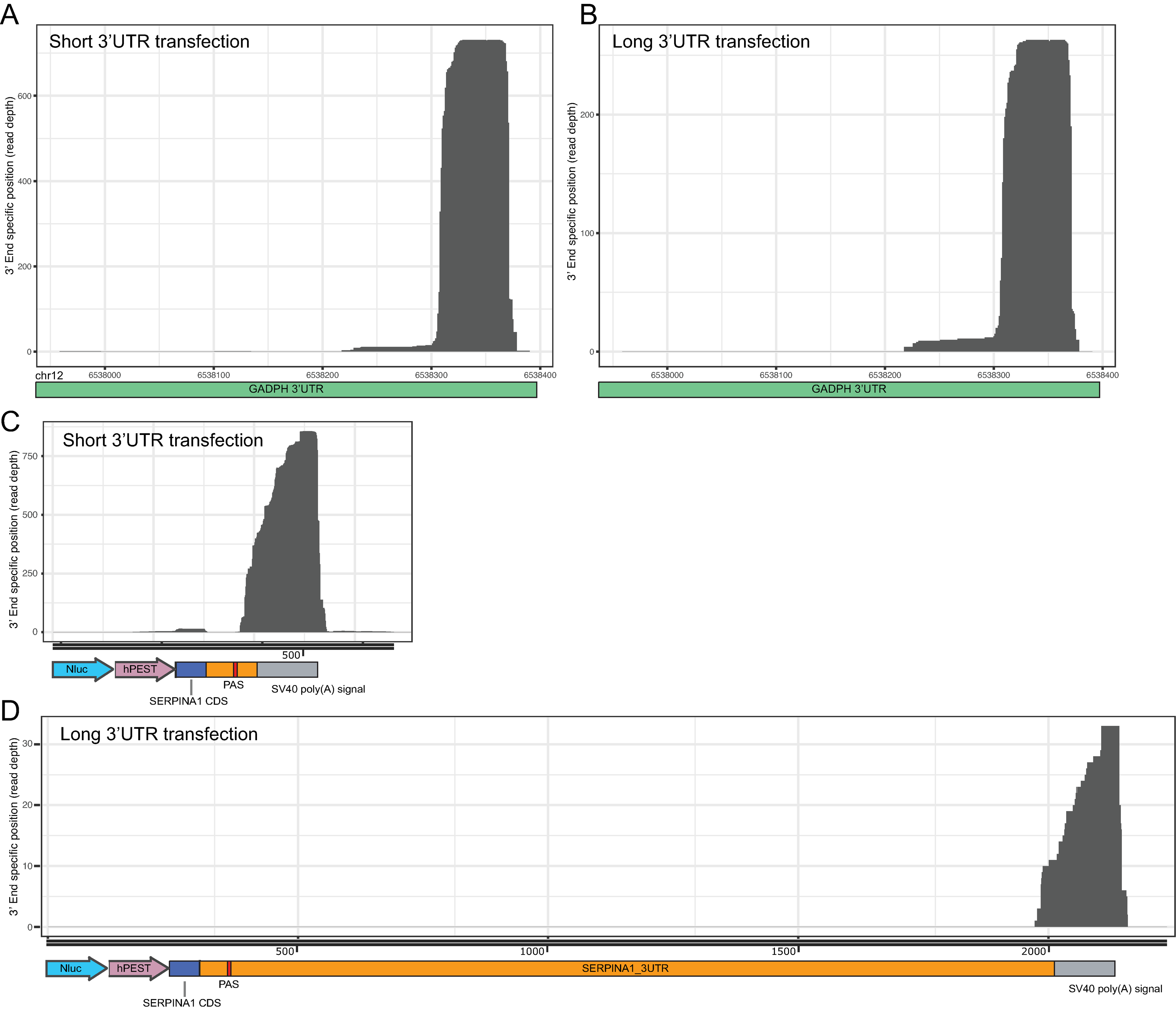

Supplement: S3 Fig — We measured 3’ end specific reads from A549 cells transfected with the nanoluciferase short or long 3’UTR construct and identified 3’ ends of endogenous and transfected SERPINA1 constructs. As expected, we found that 3’ reads cluster at the end of the 3’UTR of the endogenously expressed GAPDH in both A) short and B) long SERPINA1 3’UTR transfected cells. C) We identified a single primary 3’ end for the short 3’UTR SERPINA1 transcripts. D) We identified a single primary 3’ end for the long 3’UTR SERPINA1 transcripts at the end of the 3’UTR, mainly in the SV40 polyA signal, indicating that there is no cleavage and polyadenylation from the mutated proximal polyA site. (TIF) [file pgen.1009912.s003.tif]

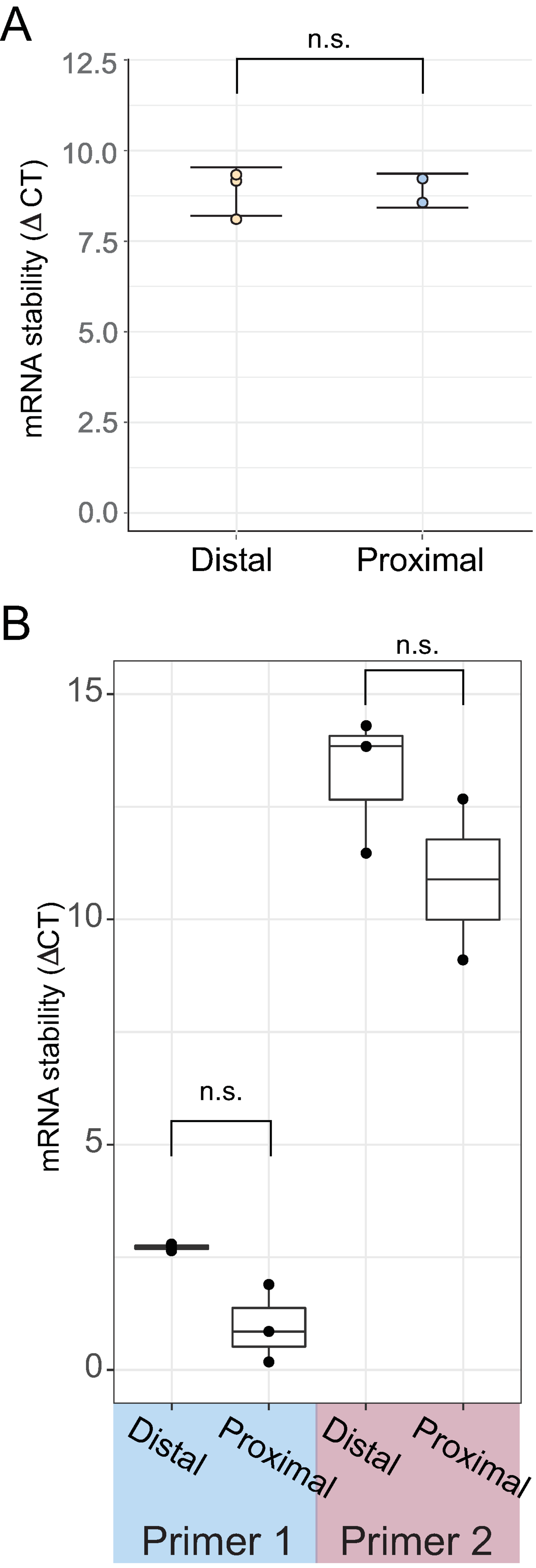

Supplement: S4 Fig — A) We performed pulse chase experiments in A549 cells using ethylene uridine (EU) and click-it chemistry for labeling with biotin-azide to measure relative mRNA stability by qRT-PCR. GAPDH was stable over the 24-hour period. We observed a steep decline in both the long and short SERPINA1 constructs, consistent with high ΔCT values, indicating similar stability for both long and short 3′ UTRs. B) We measured the levels of short and long SERPINA1 3’UTR isoforms transfected into A540 cells at equimolar amounts for steady-state RNA levels. We used two different qRT-PCR primer pairs (blue and red). We found that the short and long transcripts were present at similar levels. (TIF) [file pgen.1009912.s004.tif]

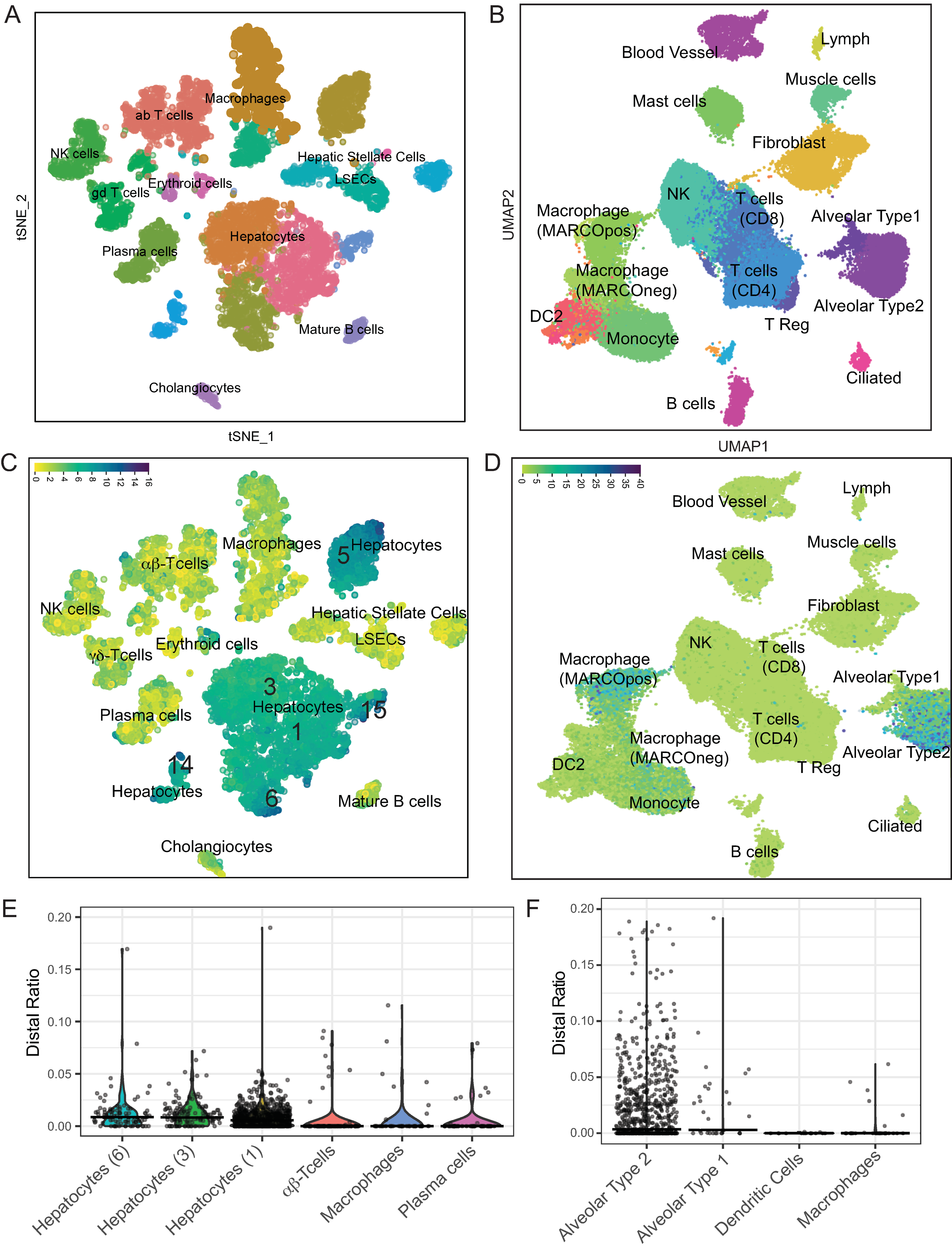

Supplement: S5 Fig — A) tSNE (t-distributed stochastic neighbor embedding) clustering of single-cell RNA-seq from five healthy human liver biopsies identifying 20 clusters as determined by [53]. B) Lung tissue from five healthy donors UMAP (Uniform Manifold Approximation and Projection) analysis of single cell RNA-seq data reveals 25 cell type Clusters as determined by [54]. C) SERPINA1 expression (normalized transcript counts) in liver cells is indicated by the yellow-green heatmap and reveals the highest counts in Hepatocyte cells, where six separate clusters were identified (clusters 1,3,5,6,14, and 15 indicated on cell map). D) SERPINA1 mRNA in lung cells is predominantly expressed in Alveolar Type 1 and 2 cells as well as Macrophages. E) Liver distal ratio distributions of SERPINA1 mRNA in single cells (indicated as open dots) for hepatocyte clusters 1,3, and 6, ab-Tcells and Plasma cells illustrates significant variation within and among cell type clusters, F) Lung distal ratio distributions in single cells indicated as dots for Alveolar Type 1 and 2, Dendritic and Macrophage cell types also indicate significant variation. (TIF) [file pgen.1009912.s005.tif]

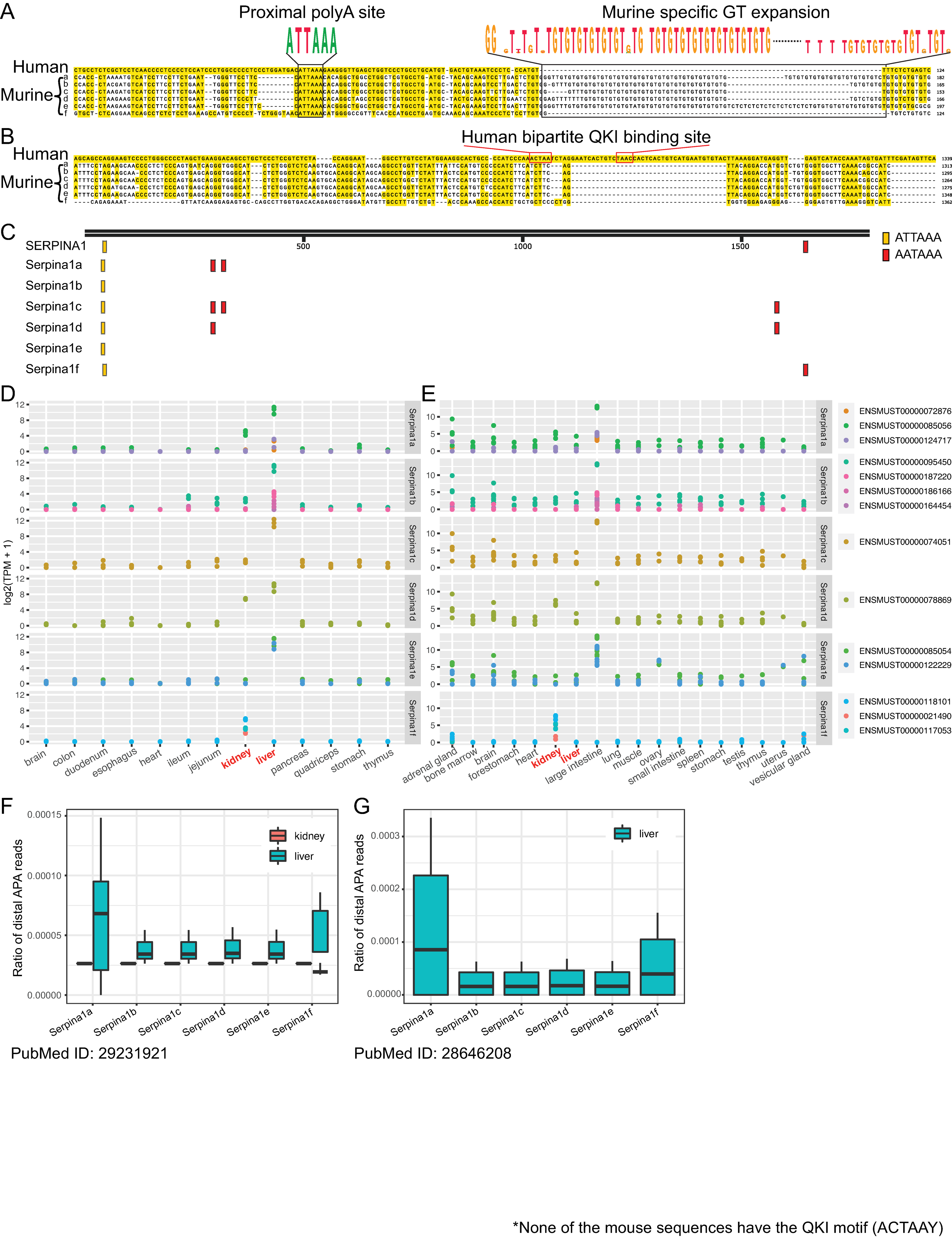

Supplement: S6 Fig — A) Alignment of human SERPINA1 and the six mouse Serpina1 paralogs (a-f) around the human proximal APA site indicating that this initial APA site is conserved in all murine Serpina1s. B) Alignment of human SERPINA1 and the six mouse Serpina1 paralogs (a-f) around the human QKI binding site illustrating poor conservation of the long 3’UTR. There are no canonical QKI bipartite or primary single QKI binding sites in the entire 3’ region (stop codon + 2kB) of murine Serpina1a-f. C) Human SERPINA1 and murine Serpina1a-f all contain a proximal APA site. Only Serpina1c, Serpina1d and Serpina1f have distal APA sites and these are not strictly conserved with the human SERPINA1 distal site. D) and E) Isoform specific alignment of Serpina1a-f from two different murine tissue datasets indicates that Serpina1a-e transcripts are highest expressed in liver tissue while Serpina1f transcripts are primarily expressed in kidney tissue. F) and G) We analyzed distal reads in Serpina1a-f in liver, and kidney tissue where available, and found no evidence for distal reads in the 3’ regions of murine Serpina1a-f. We calculated the median distal ratio to be less than 0.001 suggesting that mice do not express a long isoform of Serpina1. (TIF) [file pgen.1009912.s006.tif]
