# Supplementary material for: Alternative poly-adenylation modulates α1-antitrypsin expression in chronic obstructive pulmonary disease
Source: PLoS Genet. 2021 Nov 16;17(11):e1009912. doi: 10.1371/journal.pgen.1009912 (PMC8631626; doi:10.1371/journal.pgen.1009912)
Supplement: S1 Text — Additional methods used to generate and analyze data for supplemental figures and tables. (DOCX) [file pgen.1009912.s010.docx]

**Supplementary Methods**

**3’ end sequencing of constructs**

Total RNA was extracted from transfected A549 cells (transfection protocol in Methods) using trizol (ThermoFisher) followed by RNA column purification and on-column DNA digestion (Purelink, Invitrogen). RNA was used to create 3′ end specific libraries with the Quant Seq kit (Lexogen). Libraries were sequenced (Illumina MiniSeq, 300 kit) and the fastq sequence files used in downstream analysis. A modified Bluebee Lexogen Quant Seq FWD protocol was used for trimming, read alignment to the plasmid construct + human genome (hg38), read depth quantification and quality control steps of the Quant Seq library.

**RNA stability assays**

We performed RNA stability assays with A549 cells transfected with long or short 3′UTR nanoluciferase reporters with the Click-iT Nascent RNA Capture Kit (Invitrogen) as previously described (Methods: **RNA stability quantification)**, but at time-points 0 and 24 hours. We quantified qRT-PCR results with the Applied Biosystems QuantStudio 6. Data was analyzed in the same manner. We analyzed the amount of RNA expressed at steady-state from the short and long 3’UTR constructs by transfecting A549 cells in 6 well plates with equimolar amounts of the short and long constructs, allowed 30 hours for expression and then harvested RNA using trizol (ThermoFisher) followed by RNA column purification (Purelink Invitrogen) and DNase I digestion (Turbo DNase, Invitrogen). cDNA was synthesized with VILO (Invitrogen). qRT-PCR (PowerUp Sybr Green Master Mix, Thermo Fisher Scientific) was performed on cDNA templates with primers to amplify both the short and long constructs (Supplemental Table 2) using an Applied Biosystems QuantStudio 6. ΔCT values were calculated as the difference between SERPINA1 CT and GAPDH CT measurements from the same samples.

**Disease severity and polyA site usage in COPD patients**

We analyzed *SERPINA1* distal ratio measurements from LTRC patients in the context of disease severity metrics. GOLD severity of spirometric airflow limitation in COPD is based on reduction in FEV1 (Forced Expiratory Volume in 1 second) from a predicted normal value, with stage 2 defined as FEV1 % predicted between 50-80%, stage 3 defined as FEV1 % predicted between 30-50%, and stage 4 defined as FEV1 % predicted <30% ^1^.

**Conservation**

Murine *Serpina1a-f* sequences were downloaded from the UCSC Table Browser. The majority of constructs had short 3’UTRs as reference transcripts, so an additional 2 kB of 3’ sequence after the stop codon was included for alignment. ClustalW alignments were performed with human *SERPINA1* 3’UTR and all murine 3’ sequences using default parameters in SnapGene software (Insightful Science).

**Additional datasets**

Single cell sequencing data from five normal human livers is published in ^2^. Single cell lung data from five healthy donors is published in ^3^. We analyzed mouse RNA-seq data from two different studies analyzing gene expression across a large number of tissues ^4,5^ with Salmon to quantify isoform specific expression^6^. We derived length normalized distal expression ratios for each murine *Serpina1* paralog as previously described, aligning to mouse mm10 (Methods: **Calculation of SERPINA1 distal ratio from RNA sequencing)**.

**Supplementary References**

1. Vogelmeier, C.F. *et al.* Global Strategy for the Diagnosis, Management, and Prevention of Chronic Obstructive Lung Disease 2017 Report. GOLD Executive Summary. *Am J Respir Crit Care Med* **195**, 557-582 (2017).

2. MacParland, S.A. *et al.* Single cell RNA sequencing of human liver reveals distinct intrahepatic macrophage populations. *Nat Commun* **9**, 4383 (2018).

3. Madissoon, E. *et al.* scRNA-seq assessment of the human lung, spleen, and esophagus tissue stability after cold preservation. *Genome Biol* **21**, 1 (2019).

4. Sollner, J.F. *et al.* An RNA-Seq atlas of gene expression in mouse and rat normal tissues. *Sci Data* **4**, 170185 (2017).

5. Li, B. *et al.* A Comprehensive Mouse Transcriptomic BodyMap across 17 Tissues by RNA-seq. *Sci Rep* **7**, 4200 (2017).

6. Patro, R., Duggal, G., Love, M.I., Irizarry, R.A. & Kingsford, C. Salmon provides fast and bias-aware quantification of transcript expression. *Nat Methods* **14**, 417-419 (2017).
