# Supplementary material for: Alternative poly-adenylation modulates α1-antitrypsin expression in chronic obstructive pulmonary disease
Source: PLoS Genet. 2021 Nov 16;17(11):e1009912. doi: 10.1371/journal.pgen.1009912 (PMC8631626; doi:10.1371/journal.pgen.1009912)

We would like to thank all three reviewers and the editor for providing such careful feedback on our manuscript. We detail below specific responses to the reviewer's comments, and a marked-up version of the manuscript is attached with changes highlighted in yellow. Here we briefly summarize the major new experiments, data and changes we made to the manuscript in response to the reviewer comments.

1.) To more thoroughly support our claim that the long 3' UTR of the SERPINA1 mRNA primarily regulates A1AT translation and not mRNA stability, we now include a time-course of RNA stability and RNA steady state analysis in **Figure 3C** and **Supplemental Figure 4B**.
2.) To further establish the role of the QKI RNA binding we mutated the putative QKI binding site in the long 3' UTR (QKImutant). In addition, we added the QKI binding site to the short 3' UTR (Short+QKI) and tested both constructs in new reporter assays, the results of which are shown in **Figure 4F**. These data clearly show that the specific presence and absence of the QKI motif alters translation significantly, supporting our claim that QKI is controlling A1AT expression levels. QKI is also significantly differentially expressed in COPD lungs and changes the distal ratio of A1AT **(Figure 5)**.
3.) We performed 3' end sequencing of our reporter constructs (**Supplemental Figure 3**) and these data confirm sharp polyadenylation peaks occur at the expected sites for our long and short constructs.
4.) To clarify whether a model organism would be useful for these studies, we carried out a comparative sequence alignment of murine and human SERPINA 3' UTR sequences. While the proximal APA site is conserved in all mouse SERPINA genes (**Supplemental Figure 6A**), there is no evidence in RNA-seq experiments supporting a long 3'UTR in any Serpina1 gene in mice (**Supplemental Figure 6F and G**, distal ratio less than 10^{-3}). In addition, we found that the QKI binding site is unique to the human long 3' UTR (**Supplemental Figure 6B**).
5.) We have extensively revised the text to reflect these new data and moved several sections to the supplement which resulted in a more streamlined and focused manuscript. Changes are highlighted in the accompanying manuscript using a yellow highlight.

Reviewer #1:

The authors focus on the SERPINA1 gene, which is clinically relevant in the lung. Using computational analysis of several datasets and some experiments, the authors show that SERPINA1 is alternatively polyadenylated, which has not been reported before, that the long isoform is preferentially expressed in COPD disease, that the longer isoform leads to a reduced protein production in a reporter assay, and that various RNA binding proteins (RBPs) bind the long 3'UTR isoform, and can be predicted to be potentially relevant to the differential protein production and/or to the expression changes in COPD. Overall, the topic is of interest to the genetics community, as there are still not too many examples of interesting instances of alternative polyadenylation, and overall, both the computational and the experimental parts are performed rigorously. However, the overall conceptual advance here is modest. I do have

several concerns related to over-interpretation of the presented data, and suggestions for experiments that would make the paper more suitable for PLoS Genetics.

We thank the reviewer for their careful reading of our manuscript and appreciate their interest in this system and our work on it.

Major comments:
1. The analysis showing that the 3'UTR length does not affect RNA levels or stability is not convincing. The authors show a metabolic labeling chase experiment for 24 hr only, after which most labelled RNA is gone, and present it as evidence for no effect of the 3'UTR on RNA stability. Even if the half-life of the RNA is affected by 2- or 4-fold (e.g., from 2hr to 8hr), it would probably not be visible in this experiment. Several relevant time points should be tested to provide a quantitative estimate of the half -life for the short and the long UTR, also controlling for the different plasmid size used. The authors should measure also whether there is a change in steady-state mRNA levels.

We agree that our data does not show with certainty that mRNA stability is not affected by the long and short 3'UTR isoforms. As suggested by the reviewer, we repeated our metabolic labeling experiments at three earlier time points (0, 2 and 6 hours) (**Figure 3C**). These time-points were done with equimolar amounts of short and long constructs. We found that the stability of the long and short 3'UTR RNAs are similar, with less RNA in the long, but this decrease is not statistically significant. Our 24-hour stability assay showed similar results, which we have moved to the supplement (**Supplemental Figure 4A**). In addition, we have performed equimolar qRT-PCR steady-state RNA quantification experiments with two primer sets (**Supplemental Figure 4B**). Like our time-point stability assays, we see similar values for the long and short 3'UTR at the RNA level. These additional experiments support our claim that the long and short 3' UTRs have similar mRNA stability and that the long 3' UTR is primarily inhibiting translation, leading to the observed 50-fold decrease in luciferase activity. We have modified the text and figure legends accordingly. For example, we state (pg10, lines 213-218):

"However, for *SERPINA1*, we do not detect significant stability differences between the short and long isoforms at early or late timepoints (Figure 3C and Supplemental Figure 4A). We find that steady state levels of the long isoform are lower than steady state levels of the short isoform (Supplemental Figure 4B). However, this difference does not account for the large difference in translation between the short and long isoforms (Figure 3B). Thus, these experiments support the long isoform primarily inhibiting translation of the protein product."

2. The suggestion that QKI helps retain the SERPINA1 mRNA in the nucleus is very speculative. QKI has many possible functions, and only one of them is retention in the nucleus. The fact that the long isoform of SERPINA1 is preferentially nuclear is interesting, but can be affected by many other factors except QKI. Can the authors knock QKI down and measure subcellular localization of SERPINA1 mRNA? Otherwise, the statement should be appropriately toned down.

This is a good point, our evidence for QKI likely playing a role is now based on three findings:

1.) New luciferase assays showing that altering the QKI binding site influences translation - as suggested by Reviewer 2 (**Figure 4F**).
2.) Decrease in distal ratio in the shRNA knockdown of QKI (**Figure 5A**)
3.) eCLIP binding site in Region 3 (**Figure 4A**) and SHAPE structure analysis showing the binding site accessible for binding (**Figure 4E and G**).
4.) Order of magnitude higher distal ratio in nuclear vs. cytoplasmic fractionation experiments (Figure 4F).

We agree this evidence does not conclusively support that QKI acts through nuclear retention. A subcellular localization study with QKI shRNA is beyond our current expertise, we have adjusted the language referring to QKI to clearly state that QKI is involved in SERPINA1 translational repression, but we have not been able to fully establish this as the likely mechanism. For example, in the abstract (pg3, lines 57-59) we now state:

"We identify Quaking Homolog (QKI) as a modulator of *SERPINA1* mRNA translation and confirm the role of QKI in *SERPINA1* translation with luciferase reporter assays."

In the discussion (pg18, lines 412-416) we now state:

"Our analysis of *SERPINA1* 3'UTR translation, structure, sequence, and interactions with RNA binding proteins (Figure 4) indicate that QKI suppresses A1AT protein expression from *SERPINA1* isoforms with long 3'UTRs. These data agree with shRNA knockdown experiments where we observe a lower distal ratio in the absence of QKI. Nuclear retention is a known function of QKI in the brain and may be part of the translational inhibition of *SERPINA1* translation in liver and lung tissue [49,56]."

3. In the results presented in Figure 5, the authors reach quite strong conclusions based on overall limited data. The ENCODE shRNA data is impressive, but it only covers a limited set of RBPs, which are knocked-down to varying degrees and not completely eliminated, and the data are from liver HepG2 cells, whereas the COPD data is for lung tissue. Therefore, it is possible that there are RBPs with a stronger effect that were either not profiled by ENCODE, not sufficiently knocked down, or have a different behavior in lung cells. The conclusions of some buffering based on the number of RBPs that pass some arbitrary thresholds are over-reaching. The text should be adjusted accordingly. Since QKI appears as relevant in multiple analyses, can the authors knock it down and see how it affects their luciferase reporter results? Or the endogenous protein levels, if possible?

We address this point in three ways. First, we carried out new luciferase reporter assay experiments now presented in Figure 4F where we specifically mutated (QKImutant) the QKI binding site in the long 3' UTR and added it to the short (Short+QKI). Both experiments result in statistically significant changes in relative luminescence relative to the long and short constructs consistent with our hypothesis that QKI is controlling translation.

Second, we report in the methods section that that the RBPs used in the analysis have (pg28, lines 658-659) "a knockdown efficiency of at least 50% compared to the scrambled control for protein and RNA." For reference, we include the summary figure from the ENCODE data set at the end of this rebuttal for review. It is also important to note that the QKI shRNA knockdown experiment does lower the distal ratio, consistent with the hypothesis that it is binding to the long 3' UTR.

Third, we changed the text referring to Figure 5 and moved all the single-molecule analysis to the supplement. The reviewer is correct that the shRNA experiments were carried out in HepG2 and that we have studied A1AT expression in the lung and we add this caveat to the text, specifically (pg15, lines 348-354).

"An important caveat in interpreting the data in Figure 5 is that it is not a comprehensive analysis of all known RNA binding proteins, since we are limited on the y-axis by existing ENCODE shRNA experiments. Furthermore, the shRNA data were collected in HepG2 cells, whereas our differential expression analysis was measured in primary lung tissue. There are therefore likely additional RBPs controlling the distal ratio in the lung that this analysis cannot reveal. It is possible that a different molecular mechanism this analysis cannot reveal explains the observed lengthening in COPD lungs."

Minor comments:
1. Figure 3E – does deletion of Region1 affect the usage of the proximal site? In general, for each of the deletions, the authors should attempt to quantify the relative Distal/Proximal usage in the 3'UTR of their reporter.

We mutated the first polyadenylation site in our reporter assay to guarantee that we would get only distal site usage in that construct. This is indicated with an X on the long reporter in **Figure 3A**, but not clearly explained, we have corrected that in the text (pg9, lines 198-201):

"In our construct design, the upstream APA site (proximal) is mutated (indicated by an X in Figure 3A) so that only the correct 3'UTR isoform can be expressed. Both constructs have an additional strong SV40 polyadenylation signal to ensure that the transcripts end as expected."

Based on Reviewer #2's suggestion we also performed QuantSeq 3' end specific sequencing experiments to confirm the reporters use only the desired poly-A site. This control experiment confirms that our constructs end at the expected location. These results are shown in **Supplemental Figure 3** and described in the text (pg9, lines 201-203):

"Using 3' end specific sequencing we confirmed that we can accurately detect 3' ends (Supplemental Figure 3A and B) and that short and long 3'UTR constructs have the expected sequence (Supplemental Figure 3C and D)."

2. Figure 4A – "Region 1" is not labelled in the figure. Also, please explain the color coding of the clusters. Are these all the proteins with clusters in the ENCODE data in this region? What threshold was used to select them?

Thank you for catching the missing label in **Figure 4A**. We have corrected this issue. The color coding indicates the different RBPs that were identified in the 3'UTR of *SERPINA1*. The majority of these RBPs have more than one identified eCLIP binding site. In the methods, we explain that these are high confidence eCLIP clusters identified through the ENCODE 3 release. We now include additional information in the methods to further how these eCLIP sites were identified (pg28, lines 653-655):

"All downloaded eCLIP sites were originally identified through the described ENCODE 3 pipeline, a stringent process that takes into account experimental reproducibility and fraction of reads in peaks (FRiP) over background[33]."

3. Since in the single-cell data the authors do not observe any statistically significant changes, and as they indicate the overall power of the analysis is severely compromised by data sparseness, I don't see what this part adds to the manuscript. It can be relocated to the supplement and mentioned in the discussion section.

We agree and have moved Figure 5 C-E to the supplement as **Supplemental Figure 5**. The main purpose of this analysis is from a clinical perspective. It has yet to be published which cell types express SERPINA1 in the lung nor had their relative abundance/expression been previously analyzed. Our data do show that Alveolar Type 1 and Type 2 cells both express the long isoform, and these cells could be more accessible to therapeutic targeting since they are at the air-liquid interface. That being said, this is speculative, and we do not mention it in the text and will allow readers interested in targeting SERPINA1 mRNA to draw their own hypotheses from the analysis of this data in the supplement.

Reviewer #2:

In the study led by Lackey and colleagues, the authors continue their investigation of SERPINA1 in COPD. They demonstrate that SERPINA1 pre-mRNA is subject to APA and that this appears to change in the disease setting. They characterize this event and reveal that the long isoform is significantly less translated and retained in the nucleus. Various NGS analyses nominate QKI as a potential regulator of this process and ultimately show that SERPINA1 polyA site choice varies between cell types using single cell RNA-seq. Overall, the study is interesting and has novel aspects, but more work would be needed to recommend further consideration.

We thank the reviewer for their careful consideration of our manuscript and appreciate their interest in our work.

There are two significant weaknesses with this study that limit its impact and confound the interpretation. I note that both concerns are related to each other. First, the authors rely too heavily on retrospective analyses and need to conduct more experiments that directly test

hypotheses. There is too much reliance on correlation in patient and consortium databases. Second, the model of QKI action on SERPINA1 is not made clear throughout the text likely because of my initial concern – more experimentation is required to clarify the model. Is it that QKI does not impact SERPINA1 polyA site choice but rather binds to the long isoform while in the nucleus thereby retaining it and preventing it from getting translated? Or is it that QKI binds the long 3'UTR while getting transcribed and effects PAS choice? Could both mechanisms be occurring? These are just some of the unanswered questions that lead one to get confused as to what exactly is happening in this disease.

We agree with the reviewer that our paper does not fully elucidate the mechanism of nuclear retention by QKI, and that the evidence we present is based mostly on novel analyses of existing data sets rather than specific reporter experiments. This is partly a consequence of the pandemic; we were better able to analyze existing data sets rather than do experiments due to lab lockdowns. This has shaped the paper to its current form. We do outline below a series of responses that include substantial new experimental data that address these concerns.

Specific concerns are below:

1. The type 'M, S, or Z alleles' are not explained in the text. A reader not familiar with this literature would have no basis to understand their identity.

We apologize for the oversight; these are specific disease alleles that alpha-1 antitrypsin deficiency and lead to a genetic form of COPD. This is now clearly outlined in the accompanying text (pg8, lines 170-175).

"The most common genetic variation leading to A1AT deficiency is the *SERPINA1* Z allele[7-9]. Individuals homozygous for the S allele (SS) and heterozygous for the Z allele (MZ) may also have reduced A1AT[25,26]. Although there are many other *SERPINA1* variant alleles, most are less common or do not significantly associate with A1AT levels[6]. In addition to this genetic effect, the likelihood of an individual to have lung-related A1AT deficiency-associated diseases such as COPD is strongly influenced by environmental exposures, mainly cigarette smoke[27-29]."

2. There is a conceptual misconnect associated between analyzing HepG2 cells and COPD lung tissues. If the authors are going to use HepG2 cells as a cellular model to focus on, then shouldn't they be then more focused on analysis of cirrhosis in the liver in patients?

We agree that it would be ideal to understand *SERPINA1* regulation during cirrhosis. However, RNA-sequencing and clinical data for liver cirrhosis are not currently available in A1AT deficient individuals in sufficient numbers to power the type of analysis we present here. We do use liver cell lines (HepG2) data to analyze the effect of RBPs on *SERPINA1* distal ratio (**Figure 5**) to discover correlations. Such an analysis is not possible in model lung cell lines (such as A549), which do not express *SERPINA1* sufficiently to allow for quantitative APA analysis, and would require resources equivalent to those used in the ENCODE consortium to carry out.

Our single cell analysis reveals *SERPINA1* is primarily expressed in AT1 and AT2 cells (**Supplementary Figure 5F**), and the RNA binding protein QKI is expressed in those cells as well. Our clinical data indicates that there is a difference in APA of *SERPINA1* within the lung tissue of individuals with more severe COPD (**Figure 2**). Although traditionally SERPINA1 is thought to primarily be produced in the liver and exported to the lung, it is quite clear from recent transcriptomic profiling that lung expression is likely also playing an important role in COPD.

3. The two nano-luciferase reporters need to be confirmed that the correct PAS is being utilized. A straightforward 3'RACE should suffice. As this is a key finding in the paper, such confirmation would be essential.

This is a very good point and we have carried out 3' end specific sequencing experiments. The short 3'UTR construct and the long 3'UTR constructs end appropriately (**Supplemental Figure 3**). We apologize that the construct design was unclear. In both the long and short 3'UTRs we mutated the proximal APA site and used a plasmid with a strong SV40 polyA signal to be certain that we were testing constructs with known sequence. Our 3' end specific sequencing verifies that the long and short 3'UTRs are the intended sequence. All our deletion and mutation constructs are based on the short and long constructs and the 3' end sequencing is discussed in the manuscript (pg9, lines 198-203):

"In our construct design, the upstream APA site (proximal) is mutated (indicated by an X in Figure 3A) so that only the correct 3'UTR isoform can be expressed. Both constructs have an additional strong SV40 polyadenylation signal to ensure that the transcripts end as expected. Using 3' end specific sequencing we confirmed that we can accurately detect 3' ends (Supplemental Figure 3A and B) and that short and long 3'UTR constructs have the expected sequence (Supplemental Figure 3C and D)."

4. The authors datamine eCLIP data to generate a hypothesis that QKI is a trans acting factor potentially regulating translation of SERPINA1. The go on to retrospectively analyze fractionated RNA-seq data and to analyze SHAPE data to ultimately conclude that QKI is a likely an important trans factor that binds to the long 3'UTR isoform of SERPINA1 mRNA and retain it in the nucleus. While these data appear convincing, they are also completely correlative. The authors need to conduct two sets of experiments where they: 1) mutate the QKI consensus site in their nano-luc reporter to determine whether there is an effect on translation and 2) knockdown QKI to determine whether SERPINA1 long isoforms are now relocalized to the cytoplasm. Without these experiments or similar types of experiments, it is impossible to understand cause/effect of QKI to SERPINA1.

We agree with the reviewer that a luciferase assay deleting specifically the QKI binding site is valuable and have carried out this assay (**Figure 4F**). We observe higher translation efficiency in our QKI mutation construct (QKImutant) consistent with our model that suggests no single region disproportionately affects translation. In addition, we also see decreased translational efficiency when we add the QKI binding motif (Short+QKI) to the short 3'UTR isoform (**Figure 4F)**. Our results support QKI involvement in translational repression of the long SEPRINA1

isoform, particularly after performing the suggested luciferase assays. However, we agree that the link between QKI and nuclear retention as a mechanism is suggestive and beyond the scope of this work. As such, throughout the discussion we have added caveats to all text related to this, making it clear it is likely one of many mechanisms. We state (pg13, lines 289-300):

"One known function of QKI is to retain transcripts in the nucleus, which has the effect of repressing translation [49]. Thus, a majority of the *SERPINA1* long 3' UTR should be retained in the nucleus if this is the mechanism of translation suppression. When we analyze the distal ratio from cytoplasmic and nuclear poly-A selected fractions of HepG2 and HCC (Hepatocellular carcinoma) RNA-seq data [50,51] we observe a much higher proportion of distal reads in the nuclear fraction (Figure 4H). Based on the increased ratio of distal reads in the nucleus, we posit that one mechanism by which QKI could be altering translational efficiency is by retaining *SERPINA1* mRNA in the nucleus. This would effectively inhibit translation since mRNAs must be exported to the cytoplasm to be translated by the ribosome. Additional mechanisms may be involved in translational inhibition in other regions of the 3'UTR that we did not detect since the ENCODE RNA binding protein data is not a comprehensive atlas of all known RNA binding proteins. Nonetheless, our findings demonstrate the value of integrating large scale genomic data sets in investigating post-transcriptional regulation."

5. Based upon the data presented in Figure 5A, I am puzzled how the authors make their conclusion in the context of their previous conclusions 'QKI decreased expression in COPD therefore decreased the long isoform of the SERPINA1 mRNA, which would lead to higher translation efficiency of the A1AT protein'. This conclusion now suggests that QKI impacts PAS choice and that in the absence of QKI would result in more short isoform being produced to increase translation efficiency of A1AT protein. Or is it that the authors believe that when QKI expression goes down, the long isoform is less retained in the nucleus and is now free to increase translation?

This was the most challenging finding to explain. The reviewer is correct, the decrease of QKI in COPD lungs has a matching decrease in distal ratio in knockdown experiments (**Figure 5**). However, in COPD lungs we see an increase in the amount of distal *SERPINA1* reads (**Figure 2B**). We find it most likely that altered ratios of AT1 and AT2 cells, and their corresponding *SERPINA1* distal choices, influence the rise of distal *SERPINA1* in individuals with COPD. Better understanding *SERPINA1* expression is the goal of our single-cell RNA-seq data analysis (**Supplemental Figure 5**). Indeed, the lung cell types AT1 and AT2 express *SERPINA1* with different distal ratios, but these numbers are noisy. However, single-cell RNA-seq experiments in lung tissue require complicated dissociation protocols that have not been fully developed. Future work, beyond the scope of this manuscript, is needed to develop normal and COPD single cell RNA-seq datasets with sufficient depth and resolution to address these questions. In our manuscript, we find that QKI regulates translation from transcripts with the long *SERPINA1* 3'UTR (**Figure 4F**), potentially by retaining *SERPINA1* in the nucleus (**Figure 4H**) and not on polyA site selection. We identify multiple factors, including QKI, that influence *SERPINA1* regulation. In the discussion we now state (pg15, lines 353-358):

“However, our analysis in Figures 5A and 5B of expression changes in the LTRC lung cohort reveal that in COPD lungs, multiple RNA binding proteins (including QKI and CSTF2) have altered expression that, in the context of corresponding shRNA knock-down experiments, will lead to a decreased distal ratio. This may act as a molecular buffer, effectively keeping the distal ratio from increasing more.”

Reviewer #3: The manuscript entitled “Alternative poly-adenylation modulates α1-antitrypsin expression in chronic obstructive pulmonary disease” described that there is an increase in the use of a distal poly-adenylation site in primary lung tissue RNA-seq in COPD cases when compared to controls. The authors also reported that the alternative polyadenylation event involves two sites, a proximal and distal site downstream of the A1AT stop codon. They measured the distal ratio in human primary tissue short read RNA-seq data and corroborated their data results with long read RNA-seq data demonstrated a 50-fold decreased translation efficiency and A1AT expression. They also identified QKI as a specific modulator of SERPINA1 mRNA translation through nuclear retention in COPD primary lung tissue. This study revealed a complex post-transcriptional mechanism that regulates alternative polyadenylation and A1AT expression in COPD and acts as a buffering mechanism for changes in cellular populations in the COPD. This is an interesting and well designed study. The findings are important and novel.

Comments:
1. In Figure 2, the authors made conclusions about the M, S and Z alleles. More information on the N numbers should be provided and better justified. In addition for the ZZ sample, how conclusions are made with N=1 as a control?

We apologize that these numbers were not clearer in the text. We have modified the text to include this information. The lowest sample sizes are in the PiZZ group (control=1 and COPD=7) and the difference in individuals with COPD with MM and ZZ alleles is not statistically significant due to this lack of data. We have clarified this in the text (pg8, lines 178-185):

“This dataset includes genotyped MM individuals with no disease (n= 210) and with COPD (n=284). Rare genotypes have proportionally lower representation in our data (MS: n=14 and n=20), (MZ: n=8 and n=16) and (ZZ: n =1 and n=7). As can be seen in Figure 2E, we observe a higher distal ratio amongst all genotypes with COPD (red box plots, Figure 2E). The distal ratio is significantly higher for individuals with COPD and an MS (p=0.005) or MZ (p=0.013) genotype compared to individuals with COPD and an MM genotype (Figure 2E). In the LTRC data set there were only n=7 ZZ homozygous individuals, limiting the statistical power of this analysis (p = 0.15), but it is clear from Figure 2E that most ZZ individuals with COPD also have a higher distal.”

2. In Figures 3B and C it is not clear how the data from the two isoforms validate the authors’ conclusions by inhibiting translation, while showing not effect on mRNA stability? The conclusions reported here should be better supported by the data.

The reviewer is correct that we see a difference in expression between the short and long 3'UTR constructs (**Figure 3B**) and additional experiments suggested by the reviewer #2 further support the inhibition of translational rather than RNA stability (**Figure 3C**). We have expanded our study of RNA stability to include a time course, as suggested by Reviewer 1. We found non-significant differences between the short and long 3'UTR isoforms at all time points studied (**Figure 3C**). In addition, we have performed steady-state measurement of RNA quantity (**Supplemental Figure 4B**) and see insignificant differences between the long and short isoforms. Thus, it really does appear that the long 3' UTR isoform is primarily inhibiting translation of the A1AT protein.

3. In Figure 4 the role and function of the identified RNA binding proteins to modulate translation efficiency in COPD model should be shown.

This is an excellent point and we have now added a Table (Supplemental Table 1) that summarizes the known functions of the RBPs with eCLIP binding sites in the *SERPINA1* 3'UTR. We have integrated this in the text (pg11, lines 252-254):

"While other RNA binding proteins may have a role in regulating *SERPINA1* mRNA or A1AT expression[36-38] (Supplemental Table 1), QKI's distal binding position in Regions 3 and 4 in the 3'UTR is unique."

4. Moreover, in Figure 5 the data from the single cell analysis should be validated in both mRNA and protein levels to support the authors' conclusions.

The main motivation to carry out single cell analysis was to allow 3'UTR specific measurements in individual cell types. However, due to the sparse nature of single cell data, our results are inconclusive and do not allow a completely quantitative analysis of the data analogous to that we performed on our bulk RNA-seq data. As the specific cell types expressing *SERPINA1* had not been previously published, we nonetheless think these analyses will be of interest clinically. However, we agree with the reviewer that the data for our main objective, 3'UTR length in different cell types, is non-significant between cell populations. Thus, we have moved the single cell analyses to supplementary data (**Supplemental Figure 5**), and adjusted the text to (pg16, lines 370-376):

"Read coverage in single-cell RNA-seq data is significantly sparser and has higher variability than bulk RNA-seq data. We were able to visualize the distribution of distal ratios for each cell type in clusters with sufficient cells expressing *SERPINA1* mRNA. For the liver data these include three hepatocyte clusters (1, 3, and 6) as well as Macrophages, ab-Tcells and Plasma cells (Supplemental Figure 5E). In the lung, these are Alveolar Type 1 and 2, Dendritic cells and Macrophages (Supplemental Figure 5F). We observe that the distal ratio distributions are different in each cell type, but these differences are not statistically significant in these data, mostly due to very large variations observed."

5. Finally, the manuscript lacks experiments to assess the function in a COPD model to validate the proposed mechanisms. This is a major limitation.

Ideally, we would have liked use a mouse model to complement our studies. However, mice have six *Serpina1* genes (*a-f*) and expression of a long 3'UTR is not conserved (**Supplemental Figure 6**). Thus, although the proximal polyadenylation site is conserved in mice, the post-transcriptional mechanism we report on here is human specific. Development of a new model system such as a humanized SERPINA1 3' UTR containing mouse is beyond the scope of this work. We have added discussion of mouse as a model system to the text (pg18, lines 423-433):

"Interestingly, mice have six *SERPINA1* paralogs (Serpina1a-f), which have been edited out to generate a model of A1AT deficiency and emphysema[57]. While all these paralogs share a conserved proximal polyadenylation site with the human *SERPINA1* (Supplemental Figure 6A), none of the murine paralogs contain a QKI binding site anywhere in the potential 3'UTR (Supplemental Figure 6B). Only *Serpina1c*, *d* and *f* have potential distal sites (Supplemental Figure 6C). *Serpina1c* and *Serpina1d*, as most of the other murine paralogs, have their highest expression in liver, while *Serpina1f* is expressed in the kidney (Supplemental Figure 6D-E). However, there is no evidence for significant expression of the distal region in any of the murine *Serpina1* paralogs in two different RNA-Seq datasets (median distal ratio < 0.001, Supplemental Figure 6F-G). Post-transcriptional regulation of *SERPINA1* has therefore diverged between humans and mice, and testing of *SERPINA1* therapeutics aimed at modulating RNA regulation will require development of a different animal model system."

Below is included a figure from the ENCODE dataset showing the efficacy of shRNA knockdown efficiency for each RBP in response to Reviewer 1, Question 3.

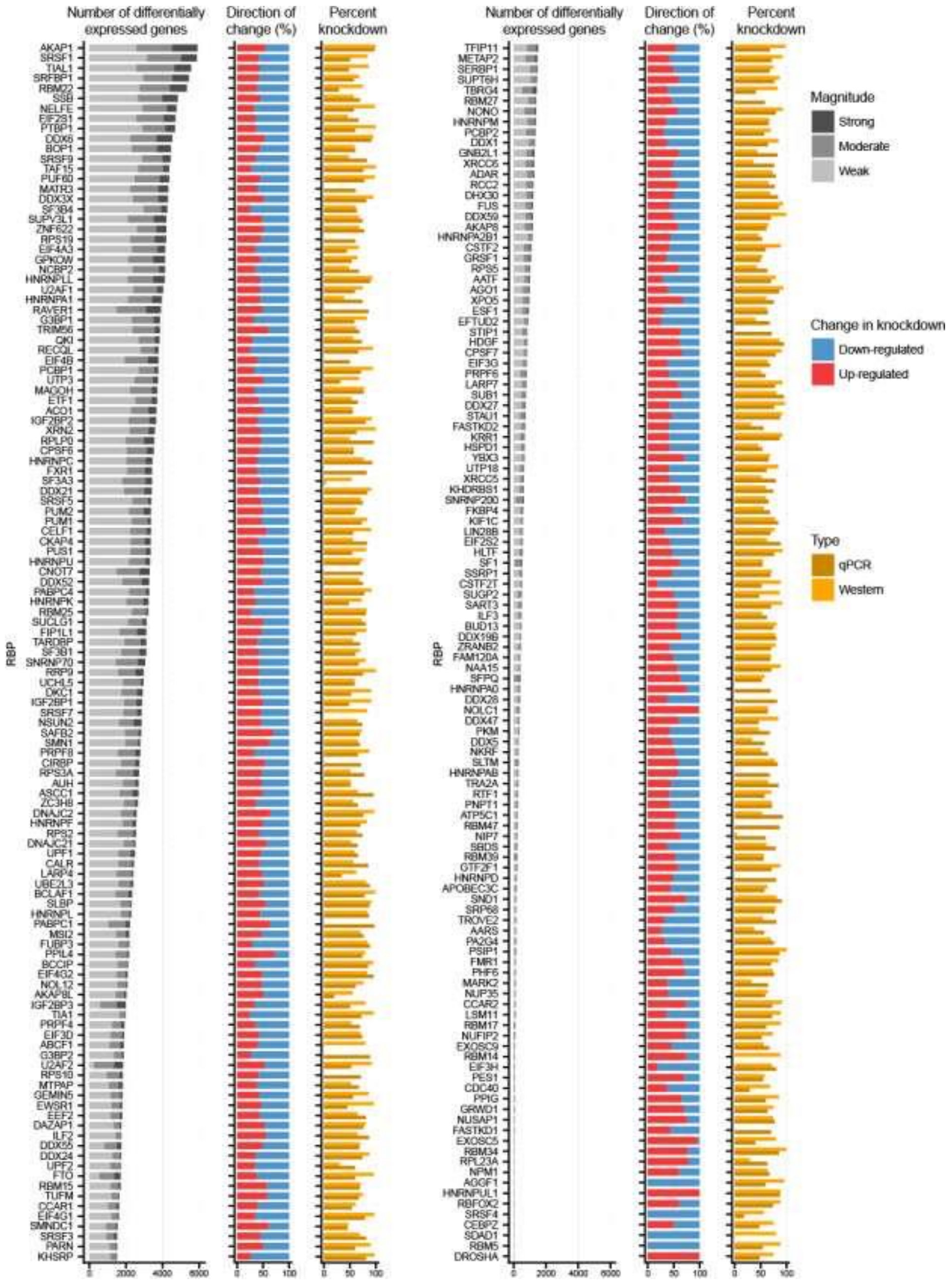

Supplement: Attachment — Submitted filename: Lackey_etal_ResponseToReviewers.pdf [file pgen.1009912.s011.pdf]
